# Translation and psychometric properties of the Persian version of the position on nursing diagnosis scale

**DOI:** 10.1002/nop2.782

**Published:** 2021-02-14

**Authors:** Tayebeh Ramezanian, Fatemeh Mohammadipour, Rasool Mohammadi, Parastou Kordestani‐Moghaddam

**Affiliations:** ^1^ Student Research Committee, Lorestan University of Medical Sciences, Khorramabad, Iran Khorramabad Iran; ^2^ Social Determinants of Health Research Center Lorestan University of Medical Sciences Khorramabad Iran; ^3^ Khorramabad Iran; ^4^ Department of Biostatistics and Epidemiology, School of Public Health and Nutrition Lorestan University of Medical Sciences Khorramabad Iran

**Keywords:** attitude, caring, nursing diagnosis, nursing process, reliability, validity

## Abstract

**Aim:**

To validate the Positions on Nursing Diagnosis scale developed by Lunney and Krenz (1992) in Persian language.

**Design:**

Cross‐sectional survey.

**Methods:**

A methodological study conducted in two stages of translation (by applying the forward and back‐translation method) and psychometric assessment was conducted in a western area of Iran. The scale was completed by 600 nurses selected by quota method from different wards of four provinces. Date of data collection is 1‐April‐2019 to 1‐Dec‐2019.

**Results:**

The Positions on Nursing Diagnosis scale showed acceptable content validity with index of 0.97. The 20 items of the Positions on Nursing Diagnosis scale load on four factors. The confirmatory factor analysis demonstrated the good fit of the model's indicators. Cronbach's alpha coefficient for the whole instrument was 0.85. The intraclass correlation coefficient was 0.86.

## 1 INTRODUCTION

While the nursing process‐based care plays a significant part in quality indicators of health care (Xiao et al., 2017), investigations have revealed that the nursing process, in practice, is either done imperfectly or not at all (Hagos et al., 2014; Lotfi et al., 2020). As a component of the nursing process, diagnosis‐based implementation provides many advantages such as significantly increased critical thinking in clinical nursing practice, and a more profound sense of professional identity and independence (Sanson et al., 2017). Furthermore, implementing nursing diagnoses can direct caregivers to improve care coordination (Lotfi et al., 2020). Although nursing diagnoses in clinical settings have a beneficial impact, underuse has been described in many countries (Akbari & Shamsi, 2011; Lotfi et al., 2020; Romero‐Sánchez et al., 2014). In developing countries, several authors have shown that nurses are unwilling to use nursing diagnosis due to time constraints, lack of nursing diagnosis experience and a biased perception in this field (Ghafourifard et al., 2012; Lotfi et al., 2020). In Iran, many studies have shown the role of attitude towards nursing diagnosis as one of the most important reasons for its application in practice (Ghanbari et al., 2017; Matbouei et al., ; Mousavinasab et al., 2020). Therefore, if nurses are positively attuned to nursing diagnosis, they use it more reliably (D'Agostino et al., 2018; Kamberi, 2019). It seems necessary, then, to perform interventions to improve attitudes towards nursing diagnoses and measure attitudes to evaluate the effectiveness of those interventions (Mousavinasab et al., 2020). However, there is no standard instrument, in Iran, to measure attitudes towards nursing diagnoses (Ghanbari et al., 2017; Matbouei et al., ).

While qualitative analysis may be a reasonable method to uncover perceptions, they cannot be clearly evaluated (Grech, 2019). Some concealed parameters are conveyed through comments; however, qualitative examination outcomes are not measurable (Collins, 2013). An option to a quantitative method is to use instruments for calculating and assessing perceptions (Vetter & Cubbin, 2019). The high value of nursing diagnoses and their significance in monitoring medical status and life‐saving factors would shift nurse perceptions about the value of nursing diagnoses and necessitate adequate instruments to attain the initial attitude (Melo et al., 2018). The Positions on Nursing Diagnosis (PND) scale can be considered an instrument in this domain. This scale includes 20 items that assess nurse attitudes about nursing diagnosis (Lunney & Krenz, 1992). This is a scale most commonly used in all clinical contexts in America, Brazil, Japan, Spain and India (D'Agostino et al., 2016).

## 2 BACKGROUND

Though psychometric properties of Positions on Nursing Diagnosis scale have been analysed in multiple languages (D’Agostino et al., 2016; Da Cruz Dde et al., 2006; Romero‐Sánchez et al., 2013), limited evidence is accessible for construct validity (Da Cruz Dde et al., 2006; Romero‐Sánchez et al., 2013). Lunney and Krenz (1992), who developed this instrument, indicated a one‐factor structure by exploratory factor analysis. Da Cruz Dde et al. (2006) analysed the Portuguese version of this scale, and a three‐factor construction was identified. The three‐factor construct was later verified by confirmatory factor analysis (CFA) (de Souza Guedes et al., 2013). Romero‐Sánchez et al. (2013) considered a single‐factor framework for the validity of the Spanish version of the Positions on Nursing Diagnosis scale in exploratory factor analysis. D’Agostino et al. (2016) also considered a single‐factor structure for the Italian version of this scale.

There is, however, no such instrument in Iran in the Farsi language. With the absence of adequate Iranian instruments, it is still possible to use the valuable Positions on Nursing Diagnosis scale, which of course needs to be translated and the psychometric properties be examined.

The Positions on Nursing Diagnosis scale is utilized in nursing diagnosis studies (Collins, 2013; D'Agostino et al., 2018; de Mattos Pimenta & da Costa Lima, 2006). In summary, as factor analysis is a method for validating the preferred instrument, it is crucial to understand the factor structures of an instrument before use in practical and clinical investigations (Kyriazos, 2018). Therefore, it was important to translate, and have validated, the Persian version of Positions on Nursing Diagnosis scale. The present research was designed in response to the following questions:

1. What are the characteristics of the Persian version of the Positions on Nursing Diagnosis scale in the Iranian population?
2. What are the psychometric properties of the Positions on Nursing Diagnosis scale in the Iranian population?

## 3 THE STUDY

### 3.1 Aim

This study aimed to validate the questionnaire in the Persian language. The objectives of the study were:

1. To translate the questionnaire into Persian language
2. To test the validity [face, content, exploratory factor analysis (EFA), and confirmatory factor analysis (CFA)]
3. To test the reliability [alpha and intraclass correlation coefficient (ICC)] of the Persian version of the Positions on Nursing Diagnosis scale.

### 3.2 Method

**FIGURE 1:**
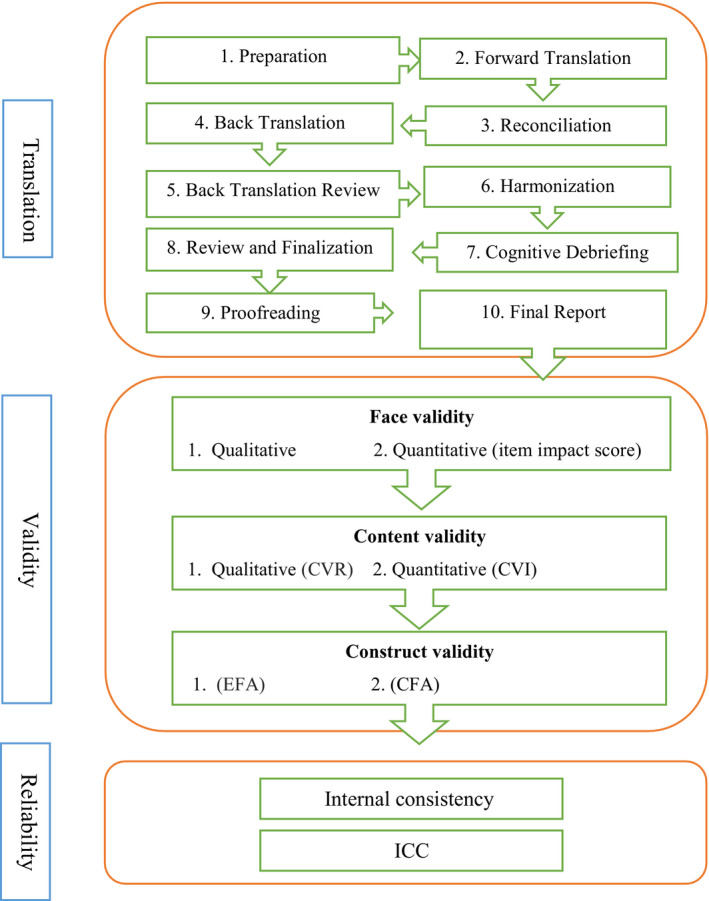
A flow chart depicting the process used to evaluating the psychometric properties

This study was a multicenter, methodological research performed in three phases in 2019–2020, including translation, assessment of validity and reliability (Figure 1). Inclusion criteria were participants who had at least bachelor degrees and consented to collaborate in the study. Exclusion criteria involved reluctance in the analysis to pursue the collaboration at any level.

#### 3.2.1 Positions on Nursing Diagnosis (PND) scale

The PND is a semantic differential scale consisting of 20 opposite adjective pairs (e.g. ambiguous/clear, helpful/hindering) that represent pole properties of the diagnostic process separated by seven short lines; each representing a number from 1–7, where “1” represents the most negative attitude and “7” the most positive. To reduce the response set, the order of positive and negative descriptors is randomized. Respondents are asked to place a mark on one of the lines between each set of adjectives. The overall score (range: 20–140) is obtained by summing the scores for each item; higher scores indicate a better attitude towards ND, and a neutral attitude is represented by a score of 80 (average score of 4 on the 20 items), and vice versa (Lunney & Krenz, 1992).

#### 3.2.2 Translation

The principles of Wild et al. (2005) were used to translate the Positions on Nursing Diagnosis scale as follows:

1. First, the questionnaire was chosen depending on study objectives during the initial phase before the translation process. The global standard and utility of this scale for nurses and students (D'Agostino et al., 2018; Romero‐Sánchez et al., 2014) and the good validity and reliability results of several reports are explanations of why this scale was selected.
2. Second, individually two translators translated the language in this stage. The first translator is from the Philippines residing in Iran having proficiency in English and a history in the medical field. The second translator was a physician with a valid certificate in the English language. A conceptual translation was sought and literal translation was avoided. The components were translated into two types: explicit and concise. Endeavours were taken to provide the general population with a clear and understandable translation.
3. Third, translations had to be integrated into a single type. In this stage, because there were minimal variations in tool translation, a panel was involved that consisted of the first and second author and the first translator, in person, and the latter translator cooperated via e‐mail by discussing both components of the two translated versions and contrasting them. Eventually, both versions were integrated into one.
4. Throughout the fourth step, a trained Persian‐English translator then translated the scale back into English (back translation).
5. The translated scale was similar to the original version.
6. The scale was provided, at this point, to a specific group of nurses to collect their cognitive knowledge to detect potential difficulties in the questionnaire, considering that the final translation had been similar to the original version. At this stage, the first researcher interviewed five nurses individually. Participants were selected based on the variables of age, sex, level of education, employment status and place of residence to represent the target population. Nurses were asked to read each item aloud in the Persian language version of the questionnaire and then express their views on the difficulty in understanding the phrases and words, the appropriateness and proper communication of the items, the possibility of ambiguity and misunderstandings of the phrases, or the inadequacy of the meanings of the words. The participants reported no ambiguous statements.
7. In the final step, all details of the translation method were documented. A translation report and the final Persian version of the "Positions on Nursing Diagnosis scale” questionnaire were submitted to the scale developer.

#### 3.2.3 Validity

##### Face validity

In order to determine the face validity, the Persian version of the Positions on Nursing Diagnosis scale was given to one matron nurse, three clinical supervisors, one head nurse, and five nurses to identify their thoughts and recommendations on comprehension, the extent of complexity of phrases, and the presence of component ambiguities (qualitative face validity).

The item impact score for each item was used to assess the quantitative face validity. A 5‐option Likert continuum was considered for each item of the scale, and the "impact score" criteria were considered. Options of “completely important,” “important,” “almost important,” “slightly important” and “unimportant” expressions were scored 5, 4, 3, 2 and 1, respectively. Using the item impact score formula (Impact Score = Frequency (%) × Importance), quantitative face validity was calculated (DeVon et al., 2007) after the scale was completed by the nurses.

##### Content validity

Based on the measurement of the content validity ratio (CVR), and relying on viewpoints of the experts, this validity was qualitatively identified. To assess the CVR, the scale was delivered to 10 nursing professors, specialists in the area of psychometric instruments, acquainted with the principle of PND. They were asked to respond to the following options based on the Likert scale: 1 = not necessary, 2 = useful, but not essential, and 3 = essential. If the CVR score was higher than 0.75 (Lawshe, 1975), the content validity of the scale was approved based on the following formula:

$\text{CVR}=\frac{\text{the number of specialists who have checked option}3‐\left(\text{the total number of specialists}/2\right)}{\text{the total number of specialists}/2}$ Quantitative evaluation of the content validity was determined using the content validity index (CVI) following the assessment and estimation of the CVR. In this analysis, ten specialists listed in the previous stage were provided with the questionnaire to express their opinions about the relevance criteria based on the 4‐option Likert scale (1 = not relevant, 2 = somewhat relevant, 3 = quite relevant and 4 = highly relevant). The content validity index for each item was then determined (Polit et al., 2007) according to the following formula:

$$\text{CVI}=\frac{\text{the Number of the specialists who have checked option}\;3\;\text{and}\;4}{\text{the total number of specialists}}$$

##### Construct validity

There are contradictory opinions about the size of the sample needed for factor analysis (Johanson & Brooks, 2010). Various reports consider at least 300, or 200, and often 100 samples to be sufficient for factor analysis (Gunzler et al., 2013). Some reports have identified 100 samples as “bad,” 200 as “reasonably well,” 300 as “well,” 500 as “quite well” and 1,000 as “fantastic” (Kyriazos, 2018). Certain researches suggest that five to ten persons per instrument term are needed (Nunnally & Bernstein, 1994).

In this phase, 600 questionnaires were distributed among nurses employed in nine teaching hospitals affiliated with each of the University of Medical Sciences in Hamedan, Ilam, Kermanshah and Lorestan (western provinces of the country). First, the proportion of samples in every province was estimated (proportional sampling) and 30 hospitals were identified in the four provinces listed. Then, the medical‐surgical wards were randomly chosen in each hospital to distribute a questionnaire between each of the nurses (cluster sampling). Researchers asked the nurses to complete the questionnaires at the end of their work shift to avoid work‐related pressures or time constraints.

In the present study, in order to investigate the construct validity of the scale, exploratory factor analysis was used because there was conflicting information about the factor structure of the research tool. After exploratory factor analysis, confirmatory factor analysis was used to prove the existence of tool factors and match with the created model.

#### 3.2.4 Reliability

For internal reliability, after collecting data, the Cronbach's alpha coefficient was determined during two steps for 600 samples from exploratory and confirmatory factor analyses.

For test–retest reliability, the scale was completed twice in a period of 14 days by 25 nurses working in different wards and was then assessed by ICC.

#### 3.2.5 Data analysis

To describe the characteristics of the participants in the study, demographic data were analysed. The face validity phase was considered as follows: If the impact score was higher than 1.5, the items were kept and chosen for further analysis (DeVon et al., 2007). To verify the content validity, the acceptable and adequate amount for the CVR score was higher than 0.75 (Lawshe, 1975) and was equal to 0.79 for CVI (Polit et al., 2007).

The factorial structure of the Persian version of the Positions on Nursing Diagnosis scale was assessed using EFA, by performing maximum likelihood estimates and varimax rotation using SPSS 22 software, following with the CFA using the Amos 25 software. Before running the factor analyses, the SPSS random split routine was used to divide the total sample into two subsamples. Subsample 1 (*n* = 300) was used to perform the EFA, and subsample 2 (*n* = 300) was used to compute the CFA in order to test the factor solution derived from the EFA. In this phase, a standardized loading estimate score of 0.40 was used as a cut‐off point for factor loadings (Black & Babin, 2019). In addition, the CFA model was assessed using the following goodness‐of‐fit criteria:

Chi‐square value (*χ* ^2^); root mean square error of approximation (RMSEA); comparative fit index (CFI); and Tucker–Lewis index (TLI).

The optimum relative chi‐square was regarded as equivalent to or below “2.” In other criteria, values higher than 0.9, 0.8–0.89 and 0.7–0.79 reveal “excellent,” “good” and “acceptable” fit, respectively. The below 0.1 values were deemed “good” to “excellent,” and the values from 0.11–0.20 were considered “acceptable” for the RMSEA (Kyriazos, 2018).

Cronbach's *α* was used to assess the internal reliability, and the ICC was used to measure stability process of the items. Acceptable values α analysis has to be higher than 0.70, according to the literature. A low value of alpha could be due to a low number of questions. When greater than 0.9, α values indicate “excellent,” values between 0.75–0.9 indicate “good reliability,” values between 0.6–0.8 indicate “acceptable reliability,” values between 0.5–0.6 indicate “poor reliability,” and values lower than 0.5 indicate “unacceptable reliability” (Black & Babin, 2019). For ICC, values <0.50 indicate “poor reliability”; values between 0.50–0.75 indicate “moderate,” values between 0.75–0.9 indicate “good reliability,” and values greater than 0.90 indicate “excellent reliability” (Koo & Li, 2016).

## 4 RESULTS

Due to the paper‐based nature of this survey and the on‐hands presence of the researcher to collect the questionnaires, 600 distributed questionnaires were fully completed. The most frequent age range of the participants was under 30, as can be seen in Table 1. As the table reveals, men had the most sampling frequency. Analysis of participant job history indicated that the majority had more than five years of experience. The surveillance of participant education revealed that most of the research classes included individuals with bachelor degrees (Table 1).

**TABLE 1:** Demographic characteristics of the participants (*n* = 600)

Variable	Category	*n* (%)
Age range (years)	<30	261 (43.50)
	31–40	161 (26.80)
	41–50	100 (16.70)
	>50	78 (13.00)
Gender	Female	315 (52.50)
	Male	285 (47.50)
Marriage status	Single	338 (56.30)
	Married	262 (43.70)
Years of work experience	<5	200 (33.30)
	5–10	117 (19.50)
	11–15	127 (21.20)
	>15	156 (26.00)
Type of employment	Formal^a^	318 (53.00)
	Contract basis^b^	118 (19.70)
	Agreement^c^	52 (8.70)
	Human resources^d^	112 (18.60)
Education	BSc	500 (83.40)
	MSc	100 (16.60)

^a^ Full‐time employment

^b^ An employee who regularly scheduled in an established position, either for 40 hr per week as a full‐time employee or for less than 40 but at least an average of four hours per week as a part‐time employee

^c^ A bargaining unit nursing position created to meet a short‐term workload need of no more than one year

^d^ A nurse who has not been assigned an full‐time equivalent status and is not regularly scheduled for any designated number of hours per pay period

The mean and standard deviation for each item is presented in Table 2.

**TABLE 2:** Position on Nursing Diagnosis (PND) items with mean score and standard deviation

Items	Mean	*SD*
01. Ambiguous‐Clear	3.32	1.25
02. Meaningless‐Meaningful	5.61	1.57
03. Pleasant‐Unpleasant	4.79	1.67
04. Strong‐Weak	4.20	1.56
05. Valuable‐Worthless	4.07	1.49
06. Negative‐Positive	4.47	1.52
07. Dumb‐Intelligent	5.00	1.64
08. Comfortable‐Uncomfortable	4.38	1.64
09. Easy‐Difficult	3.99	1.44
10. Unrealistic‐Realistic	3.74	1.55
11. Helpful‐Hindering	3.94	1.28
12. Invalid‐Valid	3.41	1.23
13. Significant‐Insignificant	3.85	1.27
14. Relevant‐Irrelevant	4.17	1.15
15. Unrewarding‐Rewarding	5.13	1.808
16. Convenient‐Inconvenient	3.72	1.08
17. Acceptable‐Unacceptable	4.91	1.20
18. Bad‐Good	4.40	1.19
19. Creative‐Routine	3.35	1.26
20. Unimportant‐Important	3.75	1.16

### 4.1 Translation

The translated questionnaires were collected by interview method during the cognitive interview phase. Viewpoints were gleaned from five nurses (including three women and two men) having a mean age of 34 and professional experience of two to fifteen years, with an average job experience of eight years, in all divisions of academic hospital environment. At this point, the nurses did not report any uncertainty or difficulty with the translation.

### 4.2 Validity

Ten nurses were given the task of assessing the qualitative and the quantitative face validity of the survey questionnaire. Their perspective on the degree of challenge, suitability, and uncertainty for each component was received and eventually accepted. All items obtained impact scores above 1.5 in this analysis. CVR provided that each item had a reasonable rate of ≥0.75. Concerning CVI, corresponding to the values gathered in the questionnaire, all questions were within the range above 0.78, and, therefore, the questionnaire face and content validity was verified.

Before the EFA, the Kaiser–Meyer–Olkin (KMO) measure of sampling adequacy was 0.859 and Bartlett test results (χ^2^ = 1,094.358, *df* = 190, *p* <.001) indicated that the number of cases was sufficient for factor analysis. Furthermore, Cronbach's alpha coefficient was measured from 300 questionnaires, which was 0.937 before assessing the EFA. The study of item analysis revealed that all item correlation coefficient was between 0.3–0.9.

EFA results showed that there were four factors explaining 68.1% of the total variance.

Table 3 displays a series of questions for each factor acquired from the EFA. There was no item elimination at this point in the standard factor load of all data greater than 0.4.

**TABLE 3:** Rotated component matrix

	Rotated component matrix				
	Description	Component			
		1	2	3	4
Item 20	Unimportant‐important	0.75			
Item 19	Creative‐routine	0.72			
Item 14	Relevant‐irrelevant	0.67			
Item 10	Unrealistic‐realistic	0.66			
Item 12	Invalid‐valid	0.55			
Item 16	Convenient‐inconvenient	0.54			
Item 13	Significant‐insignificant	0.51			
Item 4	Strong‐weak	0.48			
Item 11	Helpful‐hindering		0.73		
Item 8	Comfortable‐uncomfortable		0.67		
Item 9	Easy‐difficult		0.52		
Item 15	Unrewarding‐rewarding		0.45		
Item 1	Ambiguous‐clear		0.44		
Item 6	Negative‐positive			0.67	
Item 7	Dumb‐intelligent			0.64	
Item 5	Valuable‐worthless			0.52	
Item 3	Pleasant‐unpleasant			0.51	
Item 17	Acceptable‐unacceptable				0.72
Item 2	Meaningless‐meaningful				0.56
Item 18	Bad‐good				0.55

**FIGURE 2:**
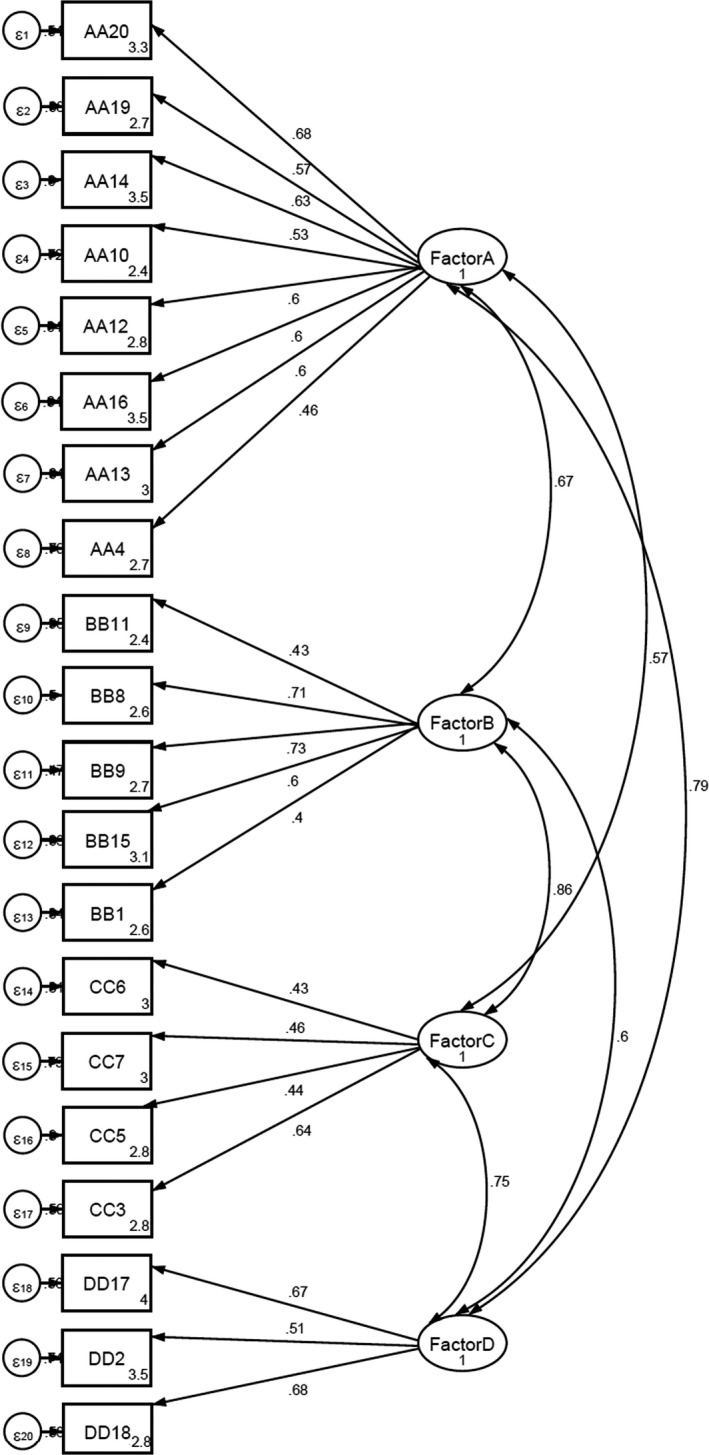
Model fitness. RMSEA = 0.06; CFI = 0.89; and TLI = 0.8

Before the CFA, the normality of the data was measured using the Kolmogorov–Smirnov test and the Q‐Q plot diagram, and since the data distribution was normal, structural equation modelling (*SEM*) was used. In CFA, four conceptual factors were components of the model of that scale (Figure 2). Goodness‐of‐fit criteria were calculated, which demonstrated a good fit of the model as follows: RMSEA = 0.056, CFI = 0.893, and TLI = 0.876.

### 4.3 Reliability

The Cronbach's alpha coefficient was 0.85 for the whole scale. Table 4 shows the Cronbach's alpha for four factors. The intra‐cluster correlation (ICC), using a two‐way mixed average measures method with a 95% confidence, reached a value of 0.86. Table 5 also provides the intra‐cluster correlation of all factors, error and confidence interval. Based on the ICC results, we concluded that the test–retest reliability of this scale is “good.”

**TABLE 4:** Cronbach's alpha coefficient of the instrument after factor analysis

FACTOR	Cronbach's alpha	Interpretation
1	0.80	Good
2	0.67	Acceptable
3	0.60	Acceptable
4	0.61	Acceptable
Total	0.85	Good

**TABLE 5:** ICC coefficient of the instrument after factor analysis

Factor	Intraclass correlation	Mean ± *SD*	95% confidence interval	Error variance	*p* Value	Interpretation
1	0.79	30.07 ± 6.71	0.76–0.83	1.11	<.001	Good
2	0.70	18.70 ± 4.72	0.63–0.72	1.32	<.001	Moderate
3	0.67	18.35 ± 4.21	0.59–0.69	1.82	<.001	Moderate
4	0.64	14.81 ± 3.01	0.57–0.70	1.11	<.001	Moderate
Total	0.86	81.93 ± 14.70	0.84–0.88	1.45	<.001	Good

## 5 DISCUSSION

To the best of our knowledge, the present study is the first attempt in Iran to translate the Positions on Nursing Diagnoses scale and report its psychometric properties. Literature review showed that there is no clear or standard method to test the attitude towards nursing diagnosis in Iran. Therefore, this research aimed to explore the psychometric characteristics of Positions on Nursing Diagnoses scale translated into the Persian language. The findings of the current study suggest that the Persian version of "Positions on Nursing Diagnosis" reveals a high validity and reliability. Validity and reliability are two essential requirements for assessing any instrument. In this analysis, the CVI of all components was above 0.78, and the CVR was well above 0.75. Evaluation of the findings derived from the data review suggests the presence of construct validity about the positions on nursing diagnosis. The goodness‐of‐fit indices of the model, as a whole, show questionnaire desirability, and four factors were defined for the instrument. Owing to the cultural variations between the countries surveyed, this observation is not consistent with the findings of the single‐factor model (D'Agostino et al., 2016; Lunney & Krenz, 1992) and the three‐factor model of construct validity (Da Cruz Dde et al., 2006; de Souza Guedes et al., 2013; Souza Guedes et al., 2013). In the present analysis, the average Cronbach's alpha coefficient was 0.85, which revealed the optimum internal reliability related to items and the overall high reliability of the scale. Cronbach's alpha coefficient was dependent on the Lunney and Krenz (1992), the Romero‐Sánchez et al. (2013), and D’Agostino et al. (2016) analyses at 0.97, 0.96, and 0.82, respectively. Cronbach's alpha coefficient of subscales was also measured in the present analysis in addition to the overall reliability calculation. In this study, the reliability was also measured using a test–retest method, which was equivalent to 0.86, while it was stated to be 0.89 in the Lunney and Krenz (1992) study.

By improving attitudes about nursing diagnosis, the implementation may influence the health outcomes of patients. Considering the validation of this scale in the Iranian society, it is possible to evaluate the quality of training courses, or classes, by any intervention that is done to improve the use of nursing diagnosis. In the process of using this tool, along with items such as quality standards of the teaching‐learning process and the formation of an accreditation council, it is possible to establish and improve the quality of nursing diagnosis.

This instrument will be used as a scale to assess the efficacy of quality management measures in the nursing process under the appropriate pre‐ and post‐test circumstances.

Apart from the translation of the intended scale using the standard translation method, this study showed strength in the conducting of both exploratory and confirmatory factor analysis to examine the validity of the scale structure. In this study, only a small sample of the total population of nurses in the country were selected from the western area of the country teaching hospitals and findings may not be generalizable to the whole country and all hospitals.

## 6 CONCLUSION

The assessment of nurse attitudes is a complex and important issue. Validation of the Positions on Nursing Diagnosis scale can be the basis for rigorous studies. Conducting various quantitative researches in the field of attitudes towards nursing diagnosis can pave the way for better understanding of the factors affecting the application of nursing diagnoses and improving the quality of services to patients.

## CONFLICT OF INTEREST

No conflict of interest has been declared by all authors.

## ETHICAL CONSIDERATIONS

The Ethical Code (IR.LUMS.REC.1399.023) was received from the Ethics Committee of the Lorestan University of Medical Sciences. Written consent has been received from the instrument developer. All study participants were mindful of the aims of the research and voluntarily signed the consent form. Nurses have been ensured the privacy of data, and anonymously, the questionnaires have been filled out.

## Data Availability

The data that support the findings of this study are available from the corresponding author, [F.M.], upon reasonable request.
